# A colorimetric biosensor with infrared sterilization based on CuSe nanoparticles for the detection of *E. coli* O157:H7 in food samples

**DOI:** 10.1128/spectrum.03978-23

**Published:** 2024-07-11

**Authors:** Yulan Guo, Jinbin Zhao, Xueer Ma, Ming Cai, Shitong Liu, Chunmeng Sun, Yuyang Chi, Kun Xu

**Affiliations:** 1 School of Medicine, Hunan Normal University, Changsha, China; 2 School of Public Health, Jilin University, Changchun, China; 3 Key Laboratory of Model Animals and Stem Cell Biology in Hunan Province, School of Medicine, Hunan Normal University, Changsha, China; 4 Engineering Research Center of Reproduction and Translational Medicine of Hunan Province, Changsha, China; University of Mississippi, University, Mississippi, USA

**Keywords:** CuSe, *Escherichia coli *O157:H7, sterilization, colorimetric assay, food samples

## Abstract

It is critical to develop quick, accurate, and efficient sterilization for detecting *Escherichia coli* O157:H7 in order to prevent infections and outbreaks of foodborne illnesses. Herein, we established a colorimetric biosensor with sterilizing properties using copper selenide nanoparticles to detect *E. coli* O157:H7. The sample was mixed with magnetic nanoprobes and nanozyme probes to form a sandwich structure, and then the unbound nanozyme probes were collected by magnetic separation. Finally, the 2,2′-azino-bis(3-ethylbenzothiazoline-6-sulfonate)-hydrogen peroxide (H_2_O_2_) reporting system was added for signal amplification. The change from colorless to green can be seen with the naked eye. Under the optimal conditions, the detection range of *E. coli* O157:H7 was 10^2^–10^6^ CFU/mL, and the detection limit was 0.35 × 10^2^ CFU/mL. The total detection time was 80 minutes, which can be successfully applied to milk and mineral water. In addition, the colorimetric sensor can kill the target bacteria by irradiating it under a 980-nm laser for 5 minutes. In conclusion, this sensor is a promising tool for rapidly detecting foodborne pathogens and promptly eliminating bacteria.

**IMPORTANCE:**

*Escherichia coli* O157:H7 is a major threat to public health. At present, the detection methods for *E. coli* O157:H7 mainly include traditional bacterial culture, immunology (enzyme-linked immune-sorbent assay) and molecular biology techniques (polymerase chain reaction). These methods have the limitations of professional operation, waste of time and energy, and high cost. Therefore, we have developed a simple, fast, bactericidal colorimetric biosensor to detect *E. coli.* O157:H7. The entire process was completed in 80 minutes. The method has been successfully applied to milk and mineral water samples with satisfactory results, proving that the method is an effective method for real-time detection and inactivation of bacteria.

## INTRODUCTION

Food safety issues caused by pathogenic bacteria are becoming increasingly significant for human health (1). Among them, *Escherichia coli* O157:H7 is a major threat to public health since it is one of the most prevalent biological elements of foodborne pathogens that contaminate food and water, causing severe sicknesses such as diarrhea, hemolytic uremic syndrome, hemorrhagic colitis, and even death (2, 3). Every year, approximately 73,000 people in the United States suffer from mild or severe diarrhea caused by pathogenic bacteria (4). Therefore, developing sensitive, accurate, simple detection and timely sterilization for *E. coli* O157:H7 detection for early prevention, diagnosis, and control of food-borne illness infections and outbreaks is greatly important.

For the detection of *E. coli* O157:H7, several analytical techniques have been developed, including traditional bacterial cultures, immunology (enzyme-linked immune-sorbent assay), and molecular biology techniques (polymerase chain reaction) (5–7). Although they are regarded as the gold standard or recommended detection methods due to their exceptional analytical performance (8), they have some limitations such as professional operations, time and energy waste, and high cost (9). Therefore, it is essential to construct simple, accurate, and fast detection methods. To address the disadvantages above, numerous new biosensors such as fluorescence, surface-enhanced Raman scattering, and electrochemical have been widely developed due to their fast reaction speed, ease of use, low cost, and superior selectivity (10–12). Among them, the colorimetric biosensors have garnered a lot of attention due to their ease in signal readout and their unique ability to present results as visible colors and to rapidly record data (13).

Nanozymes have distinct benefits over natural enzymes in terms of high stability, high catalytic efficiency, low cost, and ease of synthesis (14, 15). The increasing usage of nanozymes, particularly those with peroxidase-like activity, such as copper-based nanozymes, noble metals (Au, Ag, and Pt), and composite nanomaterials (16–20), is a significant advance in colorimetric sensors. As a copper-based sulfur compound, copper selenide (CuSe) nanoparticles can modify other substances by Cu-S bonds (21–23). It has also been shown to have good peroxidase-like activity by boosting electron transfer (24, 25), allowing for naked-eye observation of results and having tremendous potential in the development of colorimetric sensors (22). Furthermore, CuSe exhibited an intense near-infrared (NIR) absorbance peak, and a large amount of heat energy is observed when light energy is converted into heat energy under NIR laser excitation (26). CuSe and its composites, in particular, have been widely exploited as an antitumor (27, 28) and antibacterial material (29, 30). For example, Wang et al. (31) injected CuSe into mice and found that after photothermal therapy, tumor weight and size were reduced by 87%. Notably, neither the photothermal treatment nor the physiological function of the mice’s regular organs had any discernible effects. Patel et al. (30) synthesized Ni-doped (5% and 10%) CuSe and Zn-doped (5 and 10%) CuSe nanoparticles which exhibited remarkable antibacterial activity. Therefore, it is an excellent candidate for photothermal sterilization.

**Fig 1:**
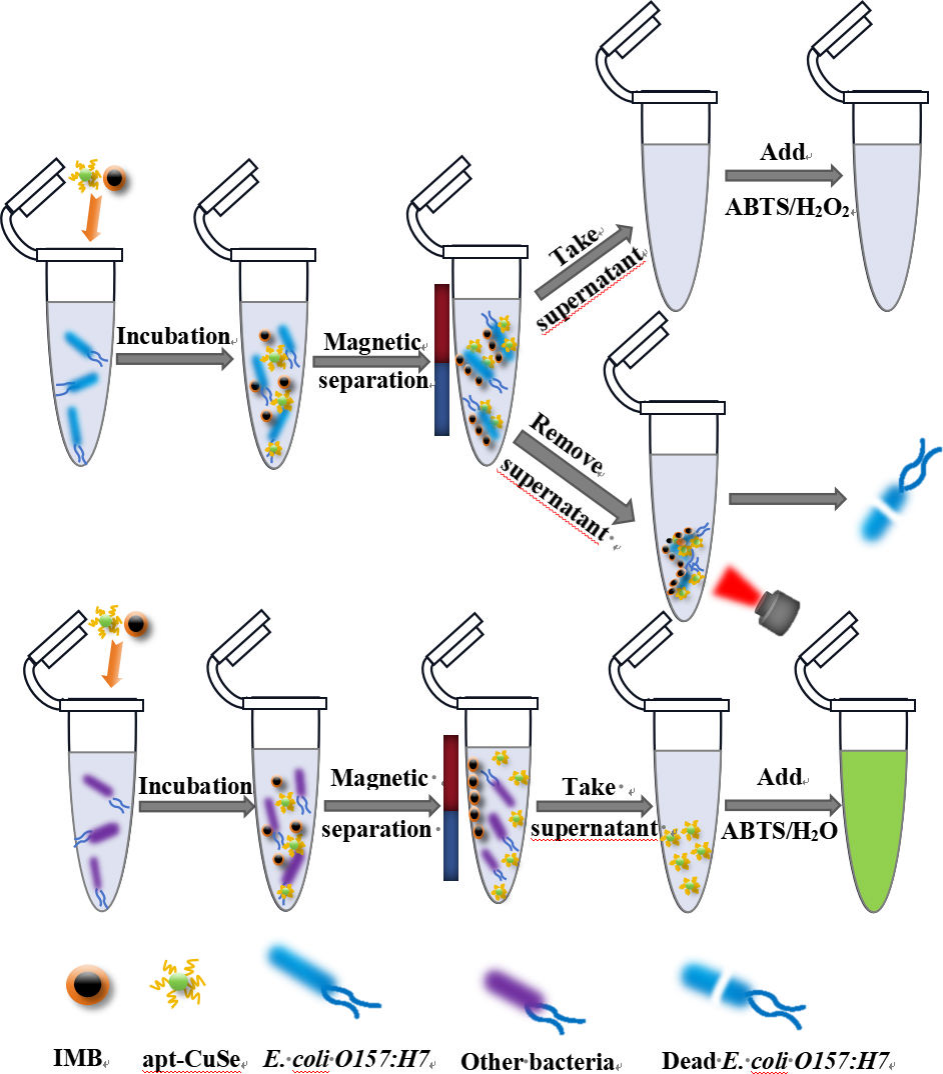
Principle of colorimetric detection method for *E. coli* O157:H7 based on CuSe.

Therefore, we developed a simple, fast, and bactericidal colorimetric biosensor to detect *E. coli* O157:H7. The complete research principle is depicted in Fig. 1. The magnetic Fe_3_O_4_ nanoparticles (MNPs) were synthesized using a co-precipitation method, and MNPs were surface-modified with specific antimicrobial peptides to identify the target bacteria, and magnetic distribution probe immunomagnetic beads (IMBs) were formed to selectively enrich and isolate the target bacteria in the sample. The aptamer (apt) was then modified on the surface of CuSe via a Cu-S bond to form the apt-CuSe nanozyme. If the sample contains *E. coli* O157:H7, IMB and apt-Cuse nanozyme can build a sandwich-type structure by binding to the surface of *E. coli* O157:H7 using dual recognition units of aptamer and antimicrobial peptides. The unbound apt-CuSe in the supernatant was collected after magnetic separation, and the 2,2′-azino-bis(3-ethylbenzothiazoline-6-sulfonate) (ABTS)-hydrogen peroxide (H_2_O_2_) reporting system was introduced for signal amplification. The color of the ABTS solution changed from green to colorless as the concentration of target bacteria in the sample increased. The color of the ABTS solution did not change considerably in samples without target bacteria. Meanwhile, the photothermal property of CuSe inactivated the target bacteria in the detection system. The method has been successfully applied to milk and mineral water samples with satisfactory results, proving that it is an effective way of detecting and inactivating germs in real time.

## MATERIALS AND METHODS

### Materials and reagents

Selenium dioxide (SeO_2_), copper sulfate pentahydrate (CuSO_4_·5H_2_O), L-ascorbic acid, H_2_O_2_, ABTS, ferric chloride hexahydrate (FeCl_3_·6H_2_O), trisodium citrate, sodium acetate (NaAc), and acetone were purchased from Beijing Chemical Reagent Co., Ltd. (China). Polyvinylpyrrolidone K-30 was purchased from Shanghai Yuanye Bio-Technology Co., Ltd. Ethylene glycol was purchased from Tianjin Fuyu (China). Tris (2-carboxyethyl) phosphine hydrochloride (TCEP) was purchased from Sigma Company (USA). Bovine serum albumin, N-(3 -(dimethylamino)-propyl)-N′-ethylcarbodiimide hydrochloride (EDC), and N-hydroxysuccinimide (NHS) were bought from Sigma-Aldrich. Phosphate buffer solution (PBS, 0.01 M, pH 7.4) was from Sangon Biotech Co., Ltd. (China). *E. coli* O157:H7 aptamer sequence was as follows: 5′-SH-(CH2)_6_-ATC CGT CAC ACC TGC TCT GTC TGC GAG GGG GGC GCG GGC GCG GCG TGG TGT TGG CTC CCG TAT, and *E. coli* O157:H7 peptide sequence was according to Wang et al.’s work (32), with the following sequence: SLLTPVP. All other reagents were analytical grade and used without further purification.

### Instruments

The UV-visible spectroscopy (UV-vis) absorption spectra were obtained using a spectrophotometer (TU-1810 DPC Persee, China). Transmission electron microscopy (TEM) images were performed on a JEOL JEM-2100F transmission electron microscope (JEOL, Japan). Fourier transform infrared (FTIR) spectra were recorded as KBr disks on a Nicolet IS5 FTIR spectrometer (Thermo Fisher Scientific, USA). SEM images were performed on a Hitachi SU8010. The dynamic light scattering (DLS) and zeta potential measurements were determined using a zeta potential analyzer (Zetasizer Nano ZS90, Malvern Panalytical).

### Synthesis of immunomagnetic nanoparticles (IMB)

First, MNPs were synthesized based on previous work. FeCl_3_·6H_2_O (1.08 g), sodium acetate (1.2 g), PEG 6000 (0.2 g), and sodium citrate (0.2 g) were dissolved in 20-mL ethylene glycol under ultrasonic conditions for complete dissolution. Then, the obtained dark red solution was then placed in a Teflon-lined stainless-steel autoclave and reacted at 200°C for 18 h. The black product was cooled to room temperature and cleaned three times with ethanol and deionized water. The MNPs were then dried for 12 h under a 60°C vacuum and kept at 4°C for future use.

Next, 1-mg/mL MNPs were activated with 10-mg EDC and 10-mg NHS dissolved in PBS buffer at 37°C for 30 minutes and washed three times by magnetic separation with PBS buffer. One hundred microliters of *E. coli* O157:H7 peptides (1 mg/mL) were added to 1-mg/mL activated MNPs. IMB was generated after an overnight incubation at room temperature, washed three times with deionized water, then resuspended with 1-mL deionized water and stored at 4°C.

### Synthesis of apt-CuSe

The CuSe was synthesized according to the previous work with some alterations. In a round-bottled flask, 3.2-mL PVP (10 mg/mL), 0.2-mL SeO_2_ (0.2 M), and 0.6-mL ascorbic acid (0.4 M) were combined for 10 minutes with magnetic agitation. Then 0.1-mL CuSO_4_·5H_2_O (0.4 M) and 0.4-mL L-ascorbic acid (0.4 M) mixed solution were added and aggressively agitated for 10 h at 30°C until the solution turned green. The green solution was collected, dialysis filtered for 24 h, and then centrifuged to remove the residue.

Apt-Cuse was synthesized as follows. Before using aptamer, the aptamer (10 µM) needed to be heated at 95°C for 5 minutes and cooled to room temperature for 2 h. Then, 1-mM TCEP was added, and the reaction was kept away from light at room temperature for 1.5 h. The 7.5-µL aptamer was mixed with 1-mL CuSe and rotated overnight at room temperature. Finally, the apt-CuSe solution was stored at 4°C for subsequent use.

### Colorimetric detection of *E. coli* O157:H7

Under optimal detection conditions, 250-µL IMB (1 mg/mL), 100-µL *E. coli* O157:H7 solution, and 50-µL apt-CuSe (100 µg/mL) were mixed, and then incubated at 37°C for 1 h. The supernatant obtained by magnetic separation was added to the ABTS-H_2_O_2_ color development system, which included 120-µL PBS (pH = 4.0), 30-mM ABTS, and 0.9-M H_2_O_2_. The reaction was carried out at 50°C for 20 minutes, and then the UV-vis spectrometer was recorded. At the same time, in order to evaluate the antibacterial effect of the developed sensor, the bacteria-IMB-CuSe precipitate was irradiated with NIR (980 nm, 1.0 W/cm^2^), and its effect was studied by plate counting.

### Detection of *E. coli* O157:H7 in real samples

To test the practicability of the proposed method, the common sterile deionized water and milk were chosen as the matrix. The samples were filtered through a 0.22-µm microfilter to collect the filtrate, and milk was centrifuged before use. Next, the filtrate was incubated with target bacteria at various concentrations. The filtrate without treatment was used as the negative control. The detection step was described in the section Colorimetric Detection of *E. coli* O157:H7, except that the sterile deionized water was changed to real samples.

## RESULTS AND DISCUSSION

### Principle

The detection principle of the method is shown in Fig. 1. MNPs were respectively modified with *E. coli* O157:H7-specific peptide via EDC and NHS coupling chemistry, which selectively enriched and rapidly separated foodborne pathogens from food substrate. CuSe functionalized with thiol-modified aptamers via Cu-S bond (apt-CuSe) was utilized as a recognition probe. First, target bacteria were captured by IMB and aptamer modified CuSe to form sandwich-type IMB-bacteria-recognition probe structures. After simple magnetic separation, the unbound apt-CuSe in the supernatant was collected and added to the color development system (H_2_O_2_ and ABTS). Due to the catalytic activity of CuSe, the color of the system showed a change from green to colorless, and the absorbance decreased at 420 nm. Therefore, the detection results of *E. coli* O157:H7 were able to be quickly discriminated by naked eyes or UV-vis spectra. At the same time, after the detection, the photothermal effect of apt-CuSe was used to inactivate pathogen bacteria.

### Characterization of IMB and apt-CuSe

**Fig 2:**
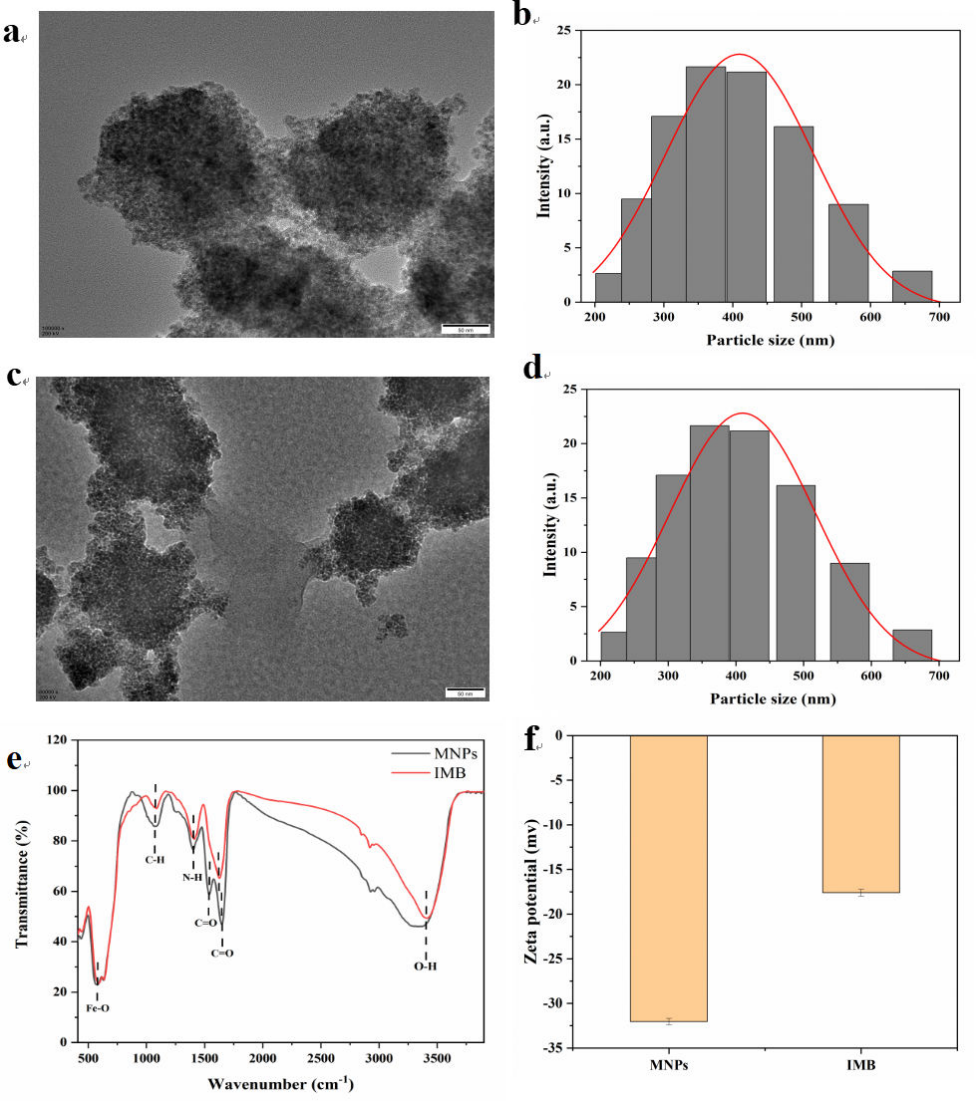
TEM photo of (**a**) MNPs and (**c**) IMB. The diameter of (**b**) MNPs and (**d**) IMB. Fourier transform infrared spectra of MNPs and IMB (**e**). Zeta potential measurements of MNPs and IMB (**f**).

TEM images clearly showed that the synthesized MNP was a typical spherical morphology with an average size of 334.6 ± 21.5 nm (Fig. 2a and b). After modification with peptides, the average diameter of IMB increased to 377.5 ± 9.7 nm, and a thin film appeared around the nanoparticle (Fig. 2c and d). The FTIR spectrum of MNPs and the IMB is demonstrated in Fig. 2e. The distinctive bands at 589, 1,066, 1,405, 1,539, 1,647, and 3,413/cm derived from the stretching vibration of Fe-O bond, C-H bond, N-H group, C=O group, C=O group, and O-H group, respectively (33). When MNPs were attached to the polypeptide, a characteristic absorption peak of 1,625.9/cmappeared. In addition, the zeta potentials of MNPs and IMB also changed, increasing from −32.04 ± 1.17 mv to −17.61 ±0.39 mv (Fig. 2f), indicating the coupling of MNPs and peptides. These results suggest that IMB was prepared successfully.

**Fig 3:**
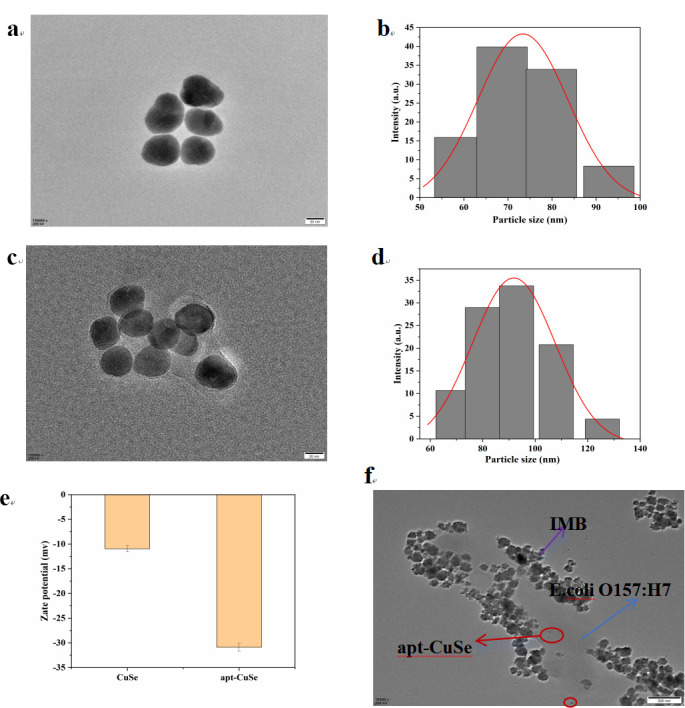
TEM images of (**a**) CuSe and (c) apt-CuSe. The diameter of (b) CuSe and (d) apt-CuSe. (e) Zeta potential measurements of CuSe and apt-CuSe. (f) TEM images of IMB-*E. coli* O157:H7-apt-CuSe probes.

Apt-CuSe was characterized by TEM, SEM, UV-vis spectroscopy, DLS, and SEM-EDS element mapping elemental analysis. TEM images, SEM images, and DLS showed that the size of CuSe was relatively uniform with an average diameter of 107.4 ± 13.5 nm (Fig. 3a and b; Fig. S1a). The co-existence and homogeneous distribution of Cu and Se elements in CuSe were demonstrated by SEM-EDS element mapping analysis (Fig. S1b and c). These results confirmed that CuSe was successfully synthesized. According to the TEM images, the modification of aptamers had no obvious effect on the morphology of CuSe, and its surfaces had a thin layer of lighter color film (Fig. 3c). The DLS result (Fig. 3d) showed the diameter of the apt-CuSe was increased to 110 ± 19.7 nm, indicating that aptamers were successfully linked to nanoparticles. Both the nanoparticles and the aptamer-modified nanoparticles were negatively charged and the zeta potentials were −10.20 ± 0.66 mv and −30.91 ± 0.88 mv, respectively (Fig. 3e), which further confirmed the successful binding of the synthesized nanoparticles to the aptamer. The UV-vis spectrum of CuSe (Fig. S2) showed an absorption peak at 994 nm, which was attributed to the surface plasmon resonance of well-dispersed CuSe. After modification with aptamer, the absorption peak of apt-CuSe was shifted to 984 nm. The obtained apt-CuSe was found to bind to the expected bacterial surface (Fig. 3f). Kinetic parameters of the reaction kinetics are measured in the supplemental data (Fig. S3; Table S2).

### Optimization of experimental conditions

**Fig 4:**
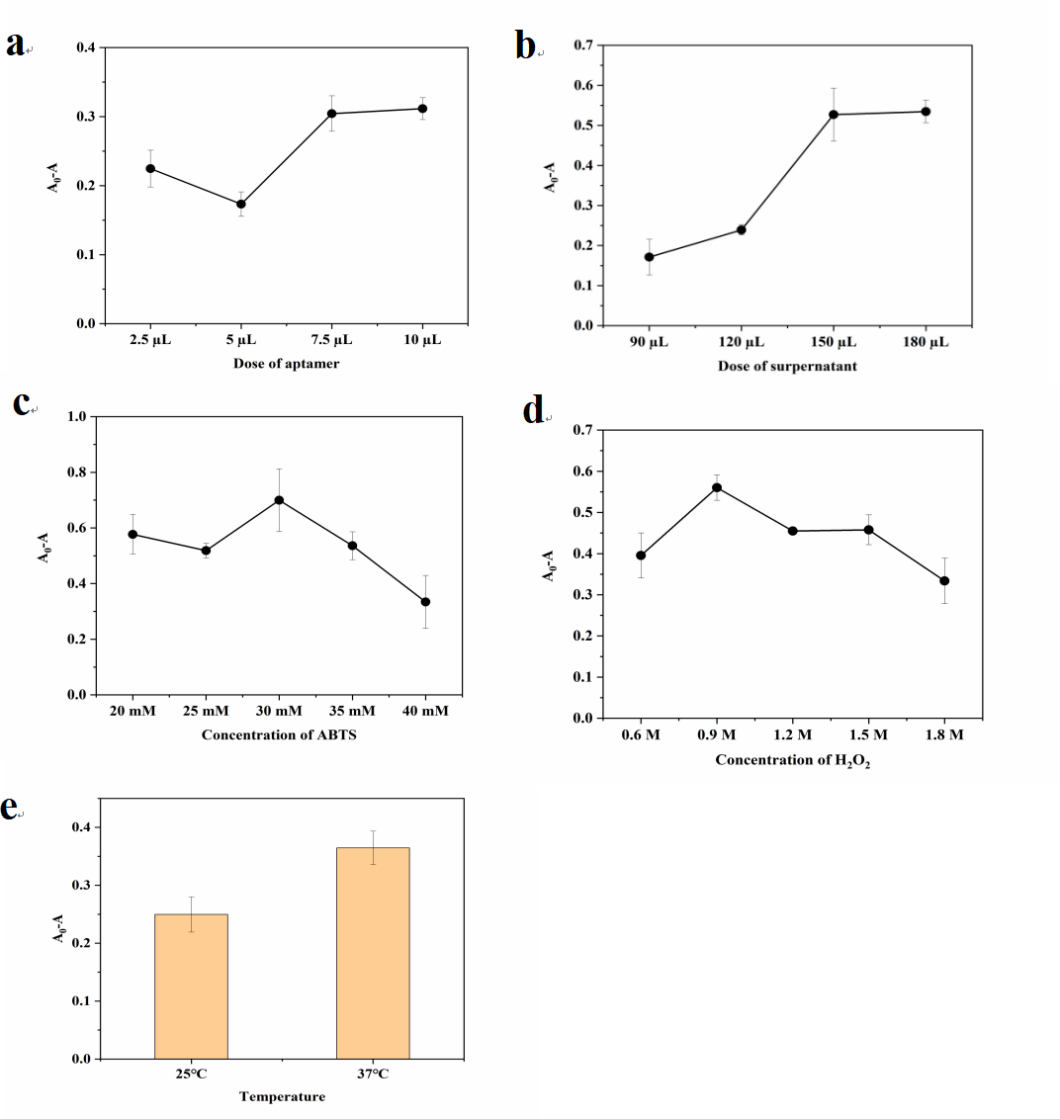
Optimization of (**a**) the dose aptamer (10 µM), (**b**) the dose of supernatant, (**c**) the concentration of ABTS, (**d**) the concentration of H_2_O_2_, and (**e**) the incubation temperature.

Several parameters were optimized to ensure detection sensitivity, including incubation temperature of target bacteria and probes, the dose of aptamer and supernatant, and the concentration of ABTS and H_2_O_2_. Ultimately, the following conditions were chosen: (i) the dose of the aptamer: 7.5 µL, (ii) the dose of the supernatant: 150 µL, (iii) the concentration of ABTS: 30 mM, (iv) the concentration of H_2_O_2_: 0.9 M, and (v) incubation temperature: 37°C. The following experiments were conducted under the aforementioned conditions (Fig. 4).

### Detection of different concentrations of *E. coli* O157:H7

**Fig 5:**
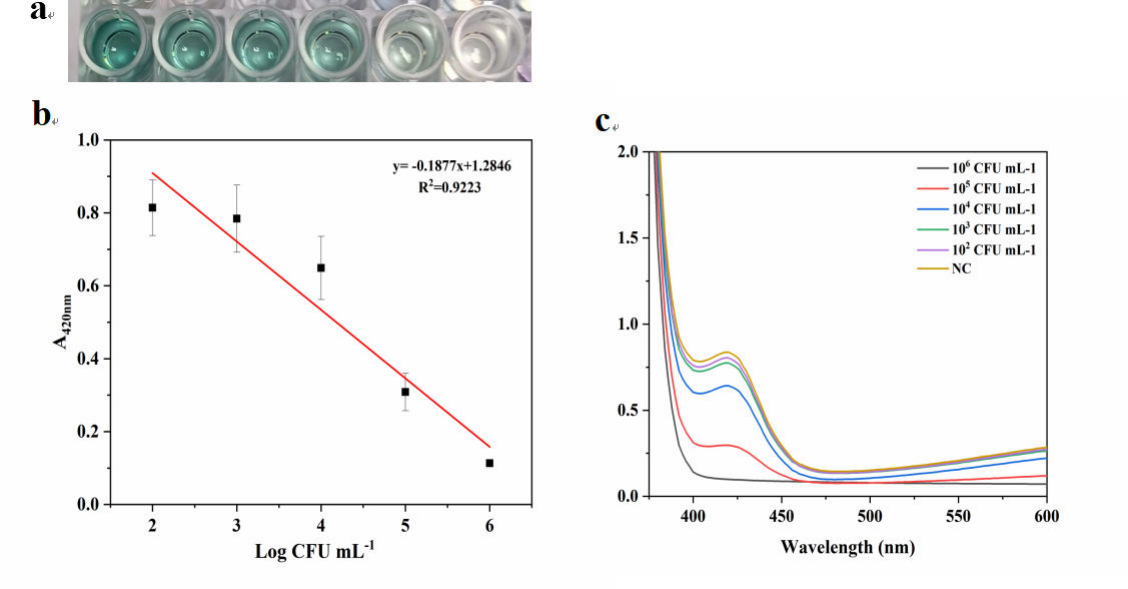
(**a**) Images of this colorimetric system with different concentrations of *E. coli* O157:H7. (**b**) Standard curve of *E. coli* O157:H7 at various concentrations (from 1 × 10^2^ to 1 × 10^6^ CFU/mL). (**c**) UV-vis spectra of *E. coli* O157:H7 at various concentrations. Error bars represent the standard deviations of three replicates.

Under optimal conditions, *E. coli* O157:H7 concentrations ranged from 10^2^ to 10^6^ CFU/mL, and sterile deionized water was used as a negative control. As shown in Fig. 5, as the concentration of *E. coli* O157:H7 increases, the color of the solution changed from green to colorless (Fig. 5a), and the absorbance at 420 nm decreased (Fig. 5b). There was a linear relationship between the concentration of *E. coli* O157:H7 and the absorbance value (Fig. 5c). The equation was *A*_420 nm_ = −0.1877 LgC + 1.2846, and the correlation coefficient was *R*^2^ = 0.9223, where *C* represents the concentration of *E. coli* O157:H7 in CFU per milliliter. The limit of detection (LOD) was 0.35 × 10^2^ CFU/mL. LOD was defined as the concentration corresponding to three standard deviations below the average absorbance of the blank control.

### Selectivity of the assay

**Fig 6:**
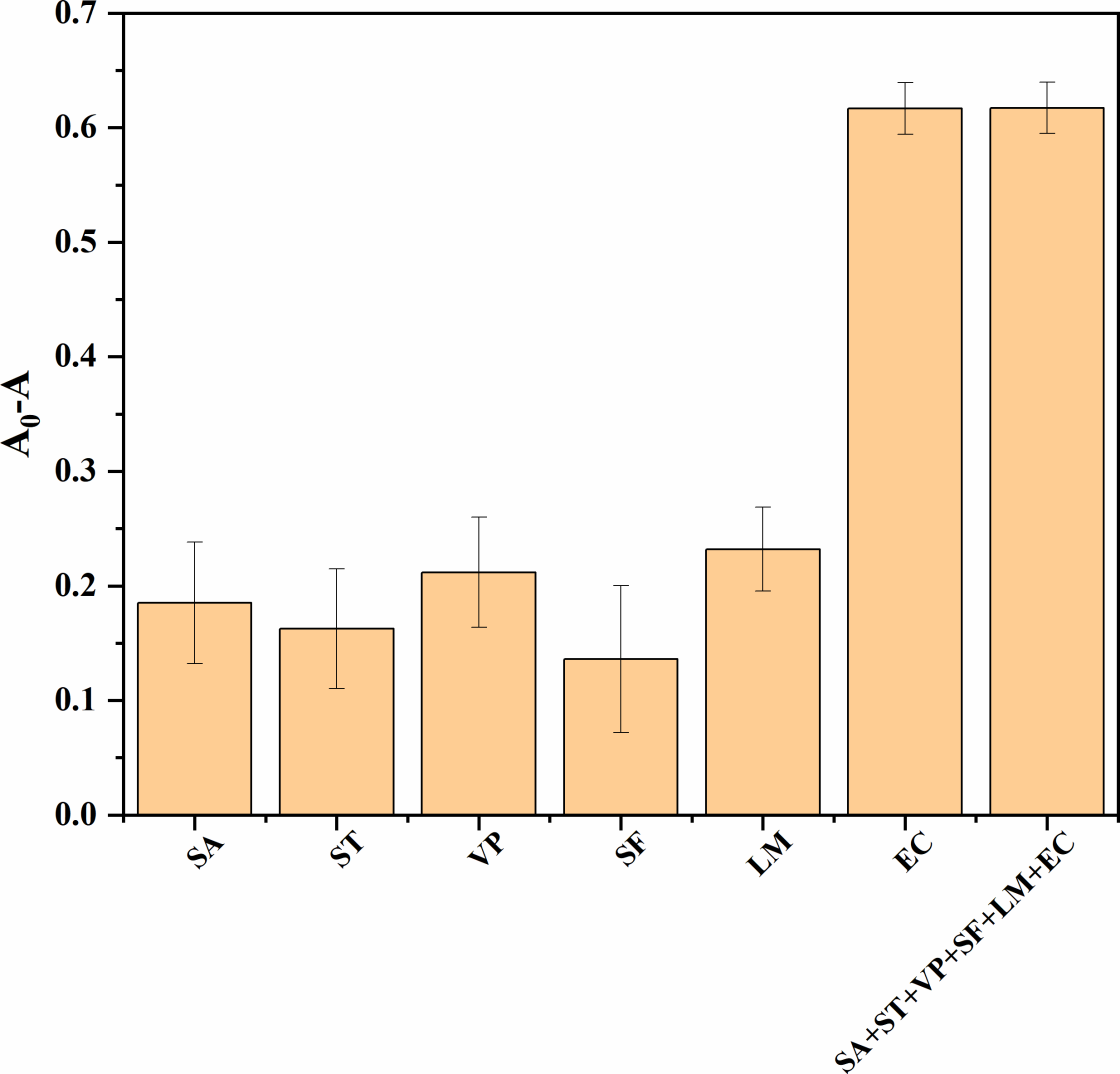
Selectivity of this detection method toward different pathogenic bacteria. EC, *Escherichia coli* O157:H7; LM, *Listeria monocytogenes*; SA, *Staphylococcus aureus*; SF, *Shigella flexneri*; ST, *Salmonella* Typhimurium; VP, *Vibrio parahaemolyticus*.

In order to test the method’s selectivity, five other common pathogens—*Salmonella* Typhimurium, *Vibrio parahaemolyticus*, *L. monocytogenes*, *S. aureus*, and *S. flexneri—*were chosen as disruptors. These bacteria were diluted to 10^6^ CFU/mL. Figure 6 shows that the absorbance difference at 420 nm grows significantly only in the presence of *E. coli* O157:H7, indicating that the detection method remains unaffected by non-target bacteria and that target bacteria can be recognized. These findings demonstrate the colorimetric sensor platform’s ability to identify and selectively collect target bacteria.

### Real sample analysis

**Fig 7:**
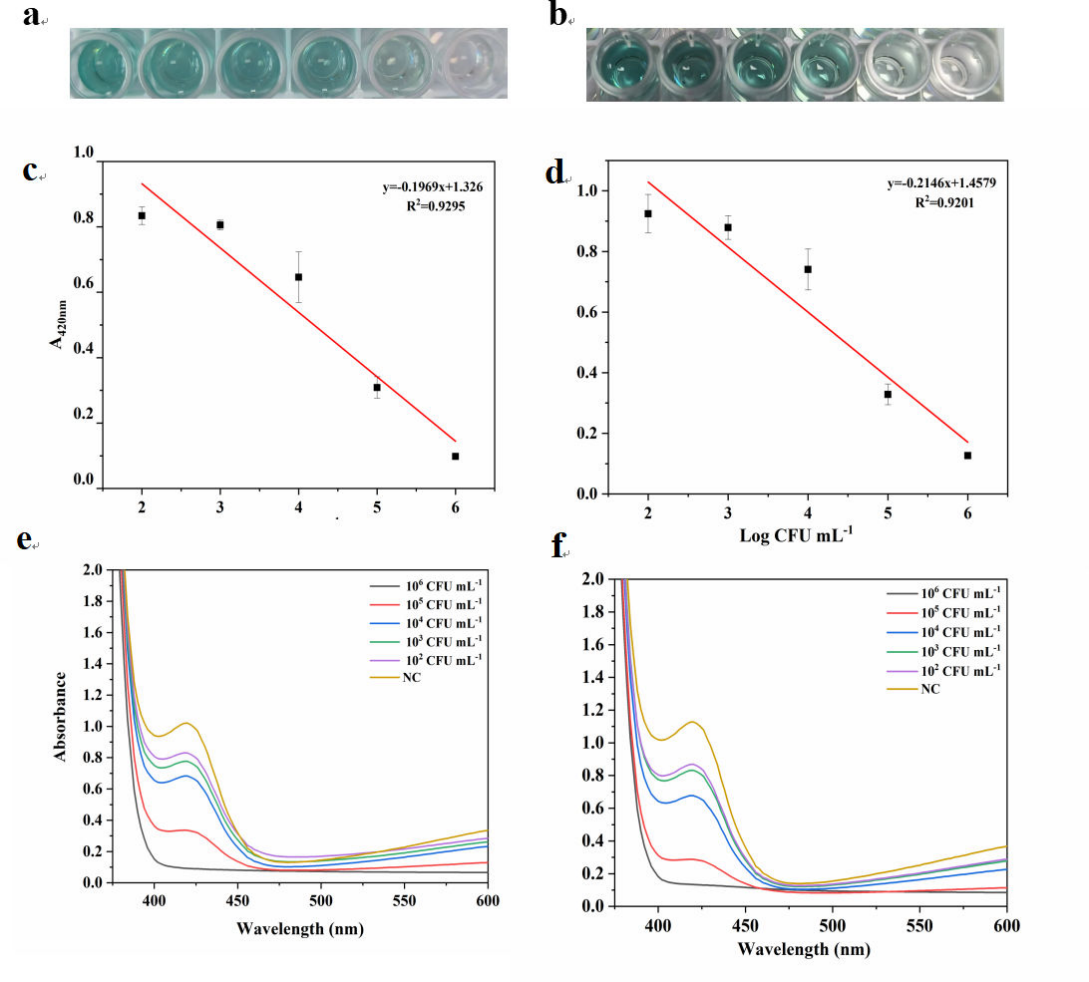
Simulated sample experiment. (**a and b**) Images of this colorimetric system with different concentrations of *E. coli* O157:H7 in the mineral water sample and the milk sample. (**c and d**) The standard curve of *E. coli* O157:H7 at various concentrations in the tap water sample and in the milk sample. (**e and f**) UV-vis spectra of *E. coli* O157:H7 at various concentrations in the tap water sample and in the milk sample.

To investigate the potential application value of this method in real samples, target bacterial cultures were diluted from 10^7^ to 10^6^, 10^5^, 10^4^, 10^3^, and 10^2^ CFU/mL using tap water and milk and then were tested with the established assay. Figure 7 shows that in the simulated sample, the absorbance difference of the sample gradually decreases with the increase of the concentration of the bacterial solution, and the difference between the colorimetric signal of the negative sample and the positive sample gradually increases. The linear equation of *E. coli* O157:H7 in mineral water and milk is as follows: the equation is *A*_420 nm_ = −0.1969 LgC + 1.326; the correlation coefficient is *R*^2^ = 0.9295; the equation is *A*_420 nm_ = −0.2146 LgC + 1.4579; and the correlation coefficient is *R*^2^ = 0.9201. The LOD is 0.36 × 10^2^ CFU/mL and 1.33 × 10^2^ CFU/mL, respectively. LOD is defined as the concentration corresponding to three standard deviations below the average absorbance of the blank control.

Besides, the detection results of our method were compared with the results of plate counting (Fig. S4 and S5). In mineral water samples, the classical method detected 260.7 ± 22 CFU/mL, and our established method detected 255.3 ± 29 CFU/mL. In milk samples, the classical method detected 119.33 ± 10 CFU/mL, and our established method detected 110.41 ± 10 CFU/mL. As shown in Table S3, the average recoveries in mineral water are between 93.97% and 103.47%. The average recoveries in milk are between 89.76% and 97.21%, and all the relative standard derivations are lower than 10%. These results illustrate that our method had a satisfactory performance in detecting real samples.

### Photothermal property and real-time sterilization of apt-CuSe

**Fig 8:**
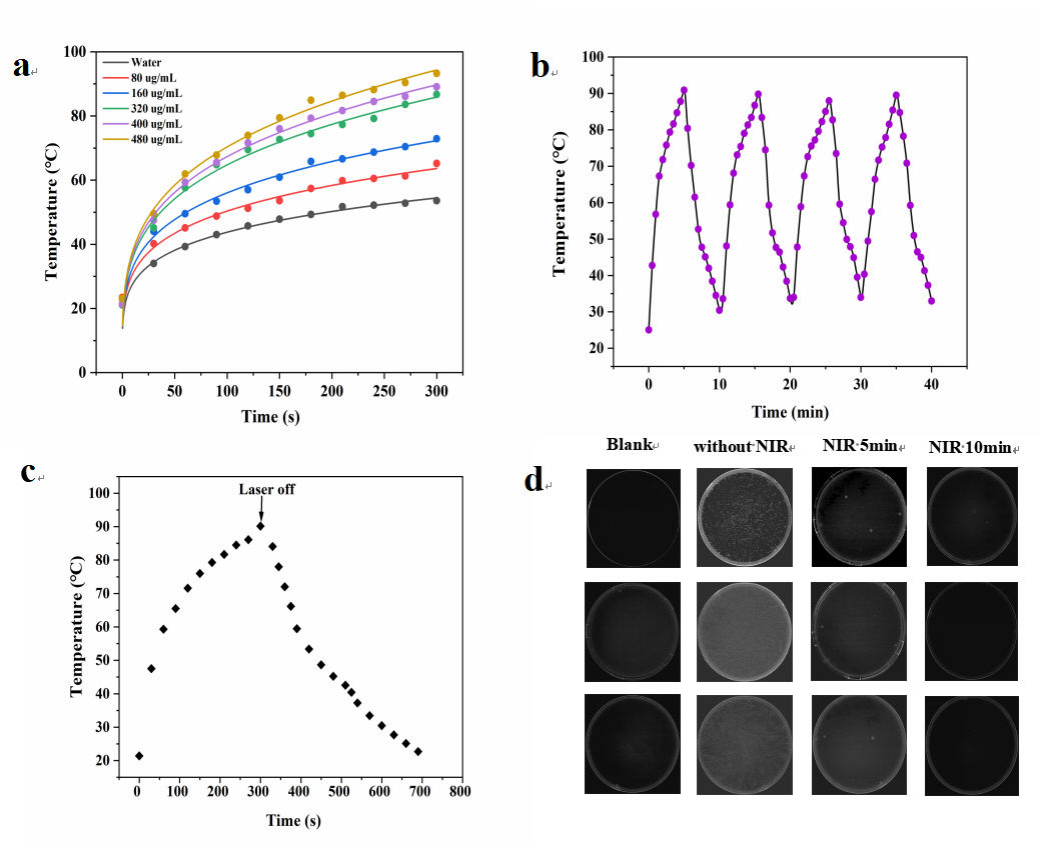
(a) Temperature elevation of different concentrations (0, 80, 160, 320, 400, and 480 µg/mL) of apt-CuSe under laser irradiation (980 nm, 1.0 W/cm, 5 minutes). (**b**) Recycling heating profiles apt-CuSe dispersion (400 µg/mL). (**c**) The photothermal conversion efficiency of apt-CuSe. (**d**) Photographs of *E. coli* O157:H7 colonies grown on Luria-Bertani (LB) agar plates before and after NIR irradiation for 5 and 10 minutes. The laser power density was 1.0 W/cm of 980-nm laser.

First, the photothermal property of apt-CuSe was verified. As shown in Fig. 8a, under the irradiation of a 980-nm laser (1.0 W/cm), the apt-CuSe solution rapidly increased from ambient temperature to more than 10°C in less than 5 minutes. Although pure water also rose, it rose far less than low-concentration, room-temperature apt-CuSe solutions, showing that light energy can be efficiently absorbed and transformed into heat energy, which is consistent with previous reports (26). The photothermal conversion efficiency calculated according to the temperature-time dynamic curve drawn (Fig. 8c) is 39.15%, which is higher than that of heavy metals and their composites, such as gold nanorods (34) (22%), Prussian blue-gold nanocomposite particles (30.77%) (35), and calcium phosphate/gold nanorods composites (38.5%) (36). There was no temperature attenuation recorded during the four on or off laser irradiation cycles (Fig. 8b), indicating outstanding photothermal stability.

CuSe surface-modified aptamers shorten the distance between the nanoparticles and the bacteria, allowing the heat energy created by the CuSe to be transferred directly to the bacteria, effectively killing the bacteria. Only a few bacteria survived after 5 minutes of irradiation, as illustrated in Fig. 8d. These results indicated that apt-CuSe can kill bacteria quickly.

### Conclusion

In this study, we successfully constructed a simple, rapid, and sterilization-enabled colorimetric biosensor based on CuSe nanozyme to detect *E. coli* O157:H7. In the presence of *E. coli* O157:H7, the unbound apt-CuSe in the supernatant can oxidize ABTS, causing the color to change from green to colorless after magnetic separation. The change in the color of the solution was related to the concentration of *E. coli* O157:H7. Meanwhile, the target bacteria in the detection system were inactivated due to the excellent photothermal effect of CuSe. The method has been successfully applied to milk and mineral water samples with acceptable results. In summary, this method is simple, specific, and low cost, and provides an effective method for timely detection and inactivation of bacteria.

## SUPPLEMENTAL MATERIAL

Supplemental material spectrum.03978-23-s0001.docx Fig. S1 to S5; Tables S1 to S3.

ASM does not own the copyrights to Supplemental Material that may be linked to, or accessed through, an article. The authors have granted ASM a non-exclusive, world-wide license to publish the Supplemental Material files. Please contact the corresponding author directly for reuse.
