# Supplementary material for: A colorimetric biosensor with infrared sterilization based on CuSe nanoparticles for the detection of E. coli O157:H7 in food samples
Source: Microbiol Spectr. 2024 Jul 11;12(8):e03978-23. doi: 10.1128/spectrum.03978-23 (PMC11302273; doi:10.1128/spectrum.03978-23)
Supplement: Supplemental material — Fig. S1 to S5; Tables S1 to S3. [file spectrum.03978-23-s0001.docx]

**Supporting Information**

**Content**

[**Figures 3**](#_Toc24825)

[**Fig.S1. (a) SEM images of CuSe. (b) and (c) SEM-EDS element mapping analysis of Cu and Se. 3**](#_Toc4873)

[**Fig. S2. the UV–Vis spectra of Cuse and apt-CuSe 3**](#_Toc19061)

[**Fig.S3. Determination of steady state kinetics of apt-CuSe nanomaterials: (a) Steady-state kinetic assay and (b) double reciprocal plots of activity of apt-CuSe. The concentration of H2O2 was 0.4 M and the ABTS concentration was varied. (c) Steady state kinetic assay and (d) double reciprocal plots of activity of apt-CuSe. The concentration of ABTS was 25 mM and the H2O2 concentration was varied. Error bars represent the standard deviation of three replicates. 4**](#_Toc3083)

[**Figure S4. Results of plate count of E. coli O157:H7 in mineral water. 5**](#_Toc23978)

[**Figure S5. Results of plate count of E. coli O157:H7 in milk. 5**](#_Toc31709)

[**Tables 5**](#_Toc9600)

[**Table S1. Bacterial strains employed in this work 5**](#_Toc9101)

[**Table. S2. Kinetic parameters of the catalytic activity of apt-CuSe and CuSe 6**](#_Toc24950)

[**Table S3. The recovery and RSD values of detecting E. coli O157:H7 in various actual samples (‾x±s, n=3) 6**](#_Toc17596)

[**Text 6**](#_Toc13399)

[**S1 Bacterial culture 6**](#_Toc2035)

[**S2 Investigation of the peroxidase-like activity of apt-CuSe 7**](#_Toc6603)

[**S3 Optimization of experimental conditions 7**](#_Toc8816)

[**1) Optimization of the dose aptamer 7**](#_Toc24933)

[**2) Optimization of supernatant dose 7**](#_Toc8071)

[**3) Optimization of the ABTS 8**](#_Toc8980)

[**S4 Photothermal property of apt-CuSe 8**](#_Toc4547)

# Figures


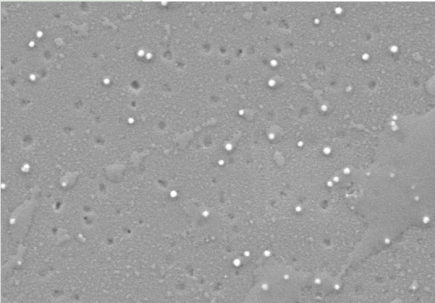

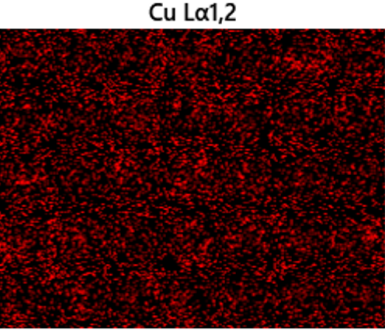

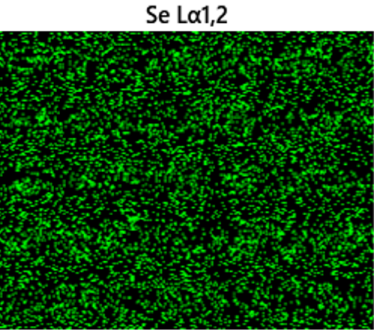


**a**

**b**

**c**

**Fig.S1.** (a) SEM images of CuSe. (b) and (c) SEM-EDS element mapping analysis of Cu and Se.





**Fig. S2.** the UV–Vis spectra of Cuse and apt-CuSe











**a**

**b**

**c**

**d**

**Fig.S3.** Determination of steady state kinetics of apt-CuSe nanomaterials: (a) Steady-state kinetic assay and (b) double reciprocal plots of activity of apt-CuSe. The concentration of H2O2 was 0.4 M and the ABTS concentration was varied. (c) Steady state kinetic assay and (d) double reciprocal plots of activity of apt-CuSe. The concentration of ABTS was 25 mM and the H2O2 concentration was varied. Error bars represent the standard deviation of three replicates.


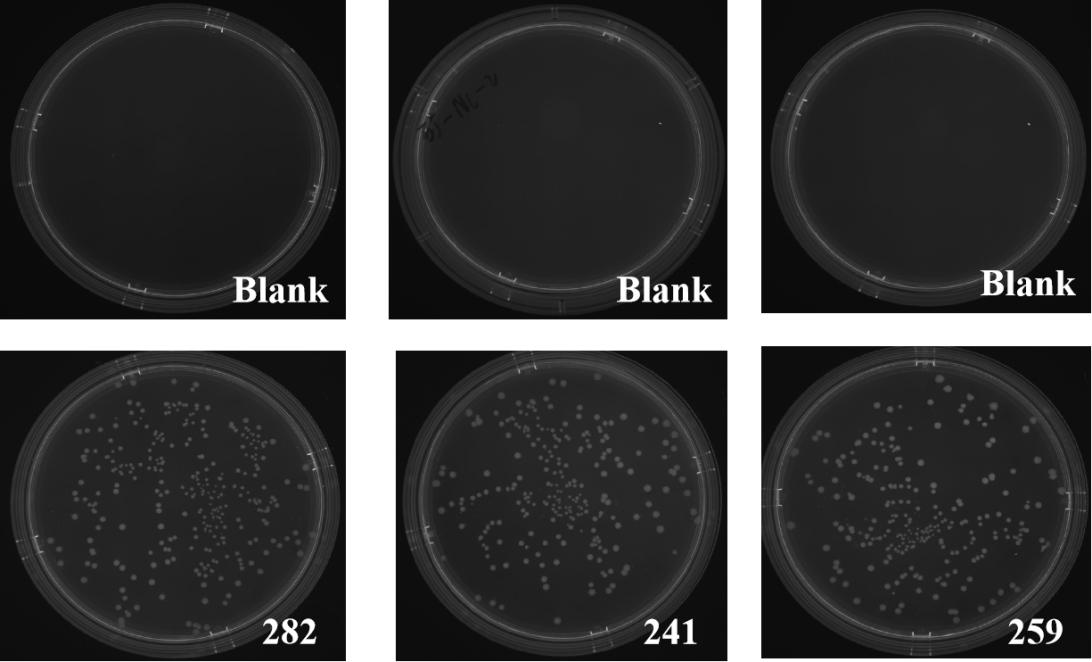


**Figure S4.** Results of plate count of E. coli O157:H7 in mineral water.


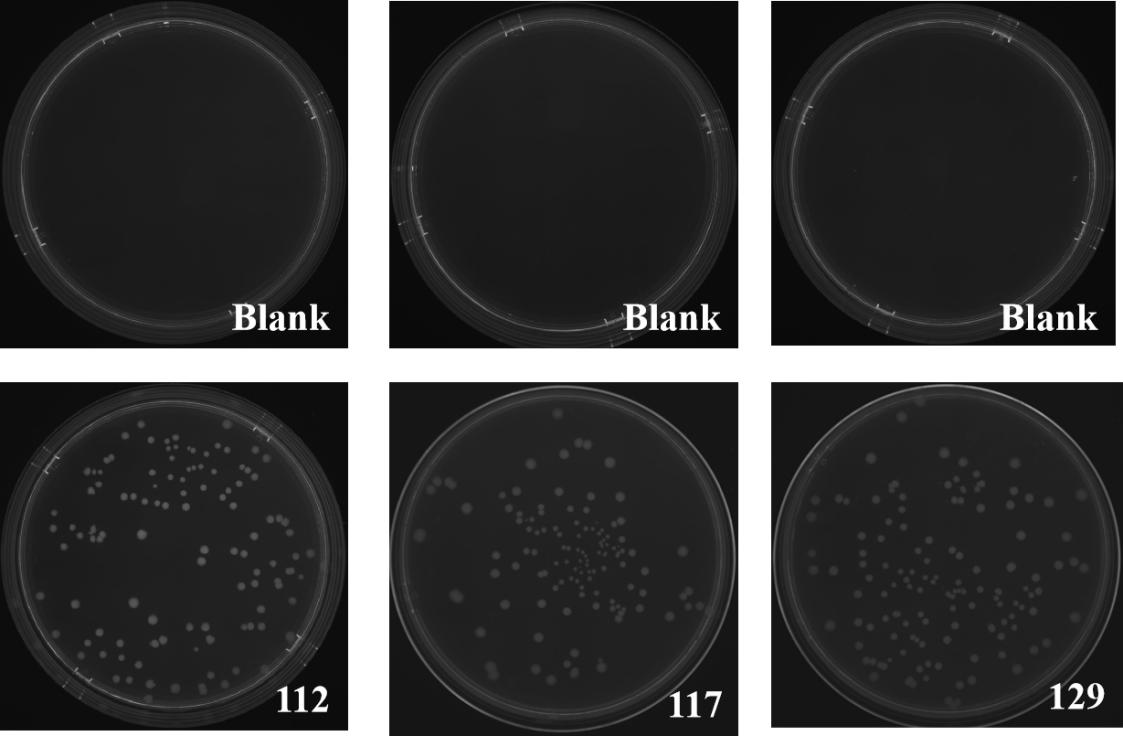


**Figure S5.** Results of plate count of E. coli O157:H7 in milk.

# Tables

**Table S1.** Bacterial strains employed in this work

| **Bacteria** | **Abbreviation** | **ATCC No.** |
| --- | --- | --- |
| *Escherichia coli O157:H7* | *E. coli O157:H7* | 35150 |
| *Staphylococcus aureus* | *S. aureus* | 25923 |
| *Salmonella typhimurium* | *S. typhimurium* | 13311 |
| *Listeria monocytogenes* | *L. monocytogenes* | 19111 |
| *Vibrio parahaemolyticu* | *V. parahemolyticus* | 17802 |
| *Shigella flexneri* | *S. flexneri* | 12022 |

**Table. S2.** Kinetic parameters of the catalytic activity of apt-CuSe and CuSe

| Catalyst | Substance | *K*m (mM) | Vmax (M/s) |
| --- | --- | --- | --- |
| apt-CuSe | H_2_O_2_ | 27.58 | 2.76×10^-8^ |
| apt-CuSe | ABTS | 0.80 | 3.02×10^-8^ |

**Table S3.** The recovery and RSD values of detecting E. coli O157:H7 in various actual samples (‾x±s, n=3)

| Samples | Plate count result (CFU/mL) | Measured (CFU/mL) | Recovery (%) | RSD (%) |
| --- | --- | --- | --- | --- |
| Mineral water | 260.7±22 | 255.3±29 | 97.90 | 4.96 |
| Milk | 119.33±10 | 110.41±10 | 92.54 | 4.07 |

# Text

**S1 Bacterial culture**

All strains of foodborne pathogenic bacteria listed in **Table S1** were obtained from the Department of Hygienic Inspection, School of Public Health, Jilin University, China.. Except for *V. parahaemolyticus*, which was revived on LA plates with 3% (w/v) NaCl, all of them were revived on Luria-Bertani agar (LA) plates. After 24 h of inoculation, a single colony of each strain was selected and cultured in the appropriate Luria-Bertani medium at 37 °C with a shaking at 180 rpm for 16 h. Lastly, the bacteria were washed three times using sterile deionized water by centrifugation at 4000 rpm for 10 minutes. Additionally, the number of bacteria was measured and expressed as CFU mL^-1^.

**S2 Investigation of the peroxidase-like activity of apt-CuSe**

The peroxidase mimic activity of the material was investigated. Firstly, 50 μL of 100 μg mL^-1^ material was added to 150 μL mixture containing PBS buffer (pH = 4.0), 25 mM ABTS and 400 mM H_2_O_2_. The sample's absorbance at 420 nm was then measured using a UV-Vis spectrophotometer. Peroxidase activity assays can be performed using an initial reaction rate, which was calculated from the molar absorption coefficient of the colorimetric substrate ABTS (ε_420 nm_ = 36,000 (M× cm)^-1^). The effects of ABTS and H_2_O_2_ concentrations in the change of reaction velocity were studied, and the kinetic parameters were calculated using the Michaelis-Menten equation.

v$\frac{V_{max}\times\left[ S \right]}{K_{m}\times\left[ S \right]}$

In the equation, v is the initial reaction velocity, v_max_ is the maximal reaction velocity, [S] is the substrate concentration and Km is the Michaelis constant.

**S3 Optimization of experimental conditions**

**1) Optimization of** **the dose aptamer**

Specific aptamers 2.5, 5.0, 7.5, and 10.0 µL of 10 µM were added to the experimental system. The results are shown in Fig. 4a. With the increase of the aptamer dose, the absorbance difference between the experimental group and the blank control group shows a trend of decreasing and increasing, reaching a plateau at 7.5 µL. At this time, the incubation temperature was 25 ℃ (room temperature), the supernatant was taken to 90 µL, the concentration of ABTS was 25 mM, and the concentration of H_2_O_2_ was 1.5 M. Therefore, specific aptamer 7.5 µL was selected for subsequent experiments.

1. **Optimization of supernatant dose**

Different supernatants (90, 120, 150, 180 µL) were added to the experimental system. The results are shown in Fig. 4b. With the increase of supernatants, the absorbance difference between the experimental group and the control group gradually increased and reached the maximum when the supernatants were 150 µL. And then it stays the same. The incubation temperature was 25 ℃ (room temperature), the specific aptamer of 10 µM was 7.5 µL, the concentration of ABTS was 25 mM, and the concentration of H_2_O_2_ was 1.5 µM. Therefore, the supernatant of 150 µL was selected for the subsequent experiment.

1. **Optimization of the ABTS**

Different concentrations of ABTS (20, 25, 30, 35, and 40 mM) were added to the experimental system, and the results are shown in Fig. 4c. With the increase of ABTS concentration, the absorbance difference between experimental group and the blank control group shows a trend of decreasing and increasing, reaching the maximum at 30 mM, and then gradually decreasing. At this point, the incubation temperature was 25 °C (room temperature), 10 µM aptamer 7.5 µL, supernatant 150 µL, and H_2_O_2_ concentration was 1.5 M. Therefore, ABTS with a concentration of 30 mM was selected for the subsequent experiment.

1. **Optimization of chromogenic substrate H_2_O_2_**

Different concentrations of H_2_O_2_ (0.6, 0.9, 1.2, 1.5, and 1.8 M) were added to the experimental system, and the results are shown in Fig. 4d. With the increase of H_2_O_2_ concentration, the absorbance difference between experimental group and the blank control group shows a trend of increased, reached the maximum value at 0.9 M, and then gradually decreased. At this point, the incubation temperature was 25 °C (room temperature), 10 µM aptamer 7.5 µL, supernatant 150 µL, and ABTS concentration of 30 mM. Therefore, H_2_O_2_ with a concentration of 0.9 M was selected for subsequent experiments.

1. **Optimization of incubation temperature**

The results are shown in Fig. 4e, the absorbance difference between experimental group and the blank control group was significantly higher than the control group at 37 °C. At this time, the specific aptamer of 10 µM is 7.5 µL, and the supernatant is 150 µL, with ABTS concentration of 30 mM and H_2_O_2_ concentration of 0.9 M. Therefore, the incubation temperature was selected at 37 °C for subsequent experiments.

**S4 Photothermal property of apt-CuSe**

Apt-CuSe (80, 160, 240, 320, 400, 480 g mL^-1^) of different concentrations prepared with deionized water were irradiated by 980 nm NIR (1.0 W cm^-1^) for 5 minutes, and the solution temperature was recorded by infrared thermal imager every 30 s. The temperature-time curve was obtained by irradiating the apt-CuSe with NIR for 5 min, then turning off the laser switch, irradiating the switch again after natural cooling for 5 minutes and the temperature was recorded every 30 s. The above operation was repeated four times. The photothermal conversion efficiency (η) was calculated with the equation:

$$\eta=\frac{hS\left( T_{max}-T_{surr} \right)-Q_{dis}}{I\left( 1-{10}^{{-A}_{980}} \right)}$$

$$hS=\frac{cm}{\tau_{s}}$$

$$\tau_{s}=\frac{t}{-ln\left( \theta\right)}$$

$$\theta=\frac{T-T_{surr}}{T_{max}-T_{surr}}$$

$Q_{dis}={hS}_{\left( water \right)}\left( T_{max\left( water \right)}-T_{surr} \right)$

Where h is the heat transfer coefficient, S is the surface area of the container, T_max_ is steady-state temperature during the heating-up period, and T_sur_ is the surrounding environmental temperature. The Q_dis_ is the heat dissipation from the light absorbed by the water and the container. *I* is the laser power, and A is the absorbance of the composite at 980 nm. C and m are the heat capacity and mass of the solution, respectively.
